# Competencies required to make use of Information Science and Technology among Japanese medical students: a cross-sectional study

**DOI:** 10.1186/s12909-024-05786-4

**Published:** 2024-08-06

**Authors:** Yuma Ota, Yoshikazu Asada, Makiko Mieno, Yasushi Matsuyama

**Affiliations:** 1 Medical Education Center, Jichi Medical University Graduate School of Medicine, Tochigi, Japan; 2 Medical Education Center, Jichi Medical University, Tochigi, Japan; 3 Center for Information, Jichi Medical University, Tochigi, Japan

**Keywords:** Medical students, Undergraduate, Information science and technology, Cross-sectional study

## Abstract

**Background:**

Competency in the use of information science and technology (IST) is essential for medical students. This study identified learning objectives and competencies that correspond with low self-assessment related to use of IST and factors that improve such self-assessment among medical students.

**Methods:**

A questionnaire was administered to sixth-year medical students across 82 medical schools in Japan between November 2022 and February 2023.

**Results:**

Three learning objectives were identified as difficult for the students to achieve: (1) provide an overview of the regulations, laws, and guidelines related to IST in medicine; (2) discuss ethical issues, such as social disparities caused by the digital divide that may arise in the use of IST in medicine; and (3) understand IST related to medical care. Further, problem-based learning, engaging with IST beyond class, and learning approach impacted the students’ acquisition of competencies related to IST. Furthermore, it was recognized that the competencies required by medical students may change over the course of an updated medical school curriculum.

**Conclusions:**

It is important for medical students to recognize the significance of learning, establishing active learning methods, and gaining experience in practically applying these competencies.

**Supplementary Information:**

The online version contains supplementary material available at 10.1186/s12909-024-05786-4.

## Background

With the development of information science and technology (IST), digital health has begun to play an important role in clinical practice [1]. The World Health Organization (WHO) has proposed digital health as an umbrella term that broadly defines the use of digital technology in health care [1]. The spread of coronavirus disease (COVID-19) has highlighted the importance of telemedicine, indicating its significance in digital health [2]. As IST is expected to continue to develop in the future, digital health will play an even greater role [3], and this will require health professionals to adapt to it [4–7].

It has been reported that physician competencies regarding eHealth and telemedicine include the appropriate use of IST based on the understanding of its benefits and risks, access and equity in digital technology, practice of patient care using IST, and ethical considerations [8, 9]. However, in a recent study on the development of digital health competencies, first- to seventh-year European medical students (*N* = 451) self-assessed competencies such as remote patient monitoring systems and the application of AI in radiology as low or very low [10]. Although many current medical students are digital natives, they lack the knowledge and skills required to utilize IST in the context of medicine [10–12].

Consequently, recent studies have highlighted the importance for medical students to acquire the competencies necessary to use IST related to digital health [11–13]. Competencies necessary to use IST in medicine and learning objectives related to the confidentiality of medical information have been included in pregraduate education in the United States, the Netherlands, Canada, the United Kingdom, and Singapore [14–18]. Additionally, it was recently reported that medical students’ digital health literacy includes skills for using electronic health records and digital ethical attitudes as well as knowledge of the advantages and disadvantages of telemedicine and medicine using advanced technologies [13]. In Japan, learning objectives related to the use of IST are described as competencies in the Model Core Curriculum for Medical Education (MCC)—an official guide for undergraduate education [19]. In the 2017 revision, only the operation of electronic health records and protection of patients’ electronic personal data were listed as subcompetencies [20]. In the 2022 revision, the use of IST was listed as one of the 10 core competencies, along with a variety of additional subcompetencies, such as the understanding of ethics and the basic principles of IST applied to medical research and medical care.

Currently, medical education in Japan focuses on the operation of electronic medical records and the protection of personal information [21, 22], while the other aspects of IST literacy education remain ambiguous. In addition, information is lacking on how students who have studied under the current curriculum self-assess their competencies to use IST, and what factors enhance their self-assessed competencies.

Therefore, we conducted a questionnaire survey among sixth-year medical students in Japan who were studying how to operate electronic medical records and protect patients’ medical information in the current Japanese curriculum. Further, we identified the factors related to self-assessment regarding the acquisition of competencies necessary to use IST. We addressed the following research questions:

Research Question 1: How do Japanese medical students under the current curriculum self-assess the acquisition of the competencies required to use IST?

Research Question 2: What factors are related to improvement in the self-assessment of the competencies that are graded as low?

Through this assessment, we expect to provide essential data for future medical curriculum management.

## Methods

### Study design

We conducted a questionnaire-based study.

### Study setting and participants

We conducted the survey from November 1, 2022 to February 28, 2023 among final year (sixth year) medical students at 82 medical schools in Japan. After graduating from high school, Japanese medical students enter medical school following completion of courses in English, mathematics, natural sciences, and social studies, but not in informatics. In medical school, students generally complete a six-year curriculum, with most completing it by November of their final year, after which, there is a period focused on preparation for the national medical examination. Given this background, we determined that this was the appropriate time to survey the acquisition of these competencies in Japan’s current medical curricula.

We used G*Power 3.1.9.6 [23] to determine the sample size, assuming a moderate effect size (f = 0.15) based on Cohen [24]. Our sample included 143 students (5% significance level), 80% power, and 16 independent variables.

### Measures

We consulted the learning objectives described as competencies in the MCC 2022 Revision to prepare the items for assessing medical students’ competencies necessary for using IST.

The MCC follows the concept of outcome-based education and identifies practical competencies that students should acquire and master by the time they graduate. The learning objectives are divided into four tiers: the first tier outlines the overview and objectives of the competencies; the second tier describes the components of each competency; the third tier, specific competencies are expressed by nouns (e.g. Knowledge of advanced IST); and the fourth tier describes the actions that learners can take and the expected outcomes. The first tier of information technology includes one item indicating IST, the second tier includes four, the third includes six, and the fourth includes 13. This curriculum is updated every three to five years after a review by medical education experts. Participants’ responses regarding the 13 items examining the learning objectives in the fourth tier (indicated by specific behaviors) were rated on a 4-point Likert scale (1 = not able, 2 = not very able, 3 = somewhat able, and 4 = able). Further, we assessed the presence or absence of information technology classes at medical schools using the Tier 3 learning objective categories to broadly capture the implementation of the classes at each university (0 = none, 1 = yes).

We used Microsoft Forms to create a questionnaire comprising three sections. The first section contained questions on sociodemographic data, such as medical students’ age, gender, type of institution (medical university or medical school of a general university), and whether they have had any work experience with IST. The second section asked about the students’ learning experiences before and after entering medical school, including whether they had learned about IST inside or outside school, when they first used IST inside or outside school, and whether they had experiences in problem-solving classes. The final section inquired about medical students’ competencies necessary to use IST. Table 1 outlines the relevance between the four-tier subcompetencies in the MCC and the question items in the present study.

**Table 1:** Correlation between MCC Four-Tier subcompetencies and Study question items

Subcompetencies in Information Technology (IT)		Question items for the current study
**IT-01: Ethics and rules for dealing with information science and technology**		
IT-01-01: Preparation for dealing with information science and technology		
IT-01-01-01	Understand the importance and social significance of using information science and technology in medicine.	**Q1**
IT-01-01-02	Understand an overview of the regulations, laws, and guidelines related to information science and technology in medicine.	**Q2**
IT-01-01-03	Discuss ethical issues, such as social disparities caused by the digital divide, that may arise in the use of information science and technology in medicine.	**Q3**
IT-01-02: Ethics and rules for using information science and technology		
IT-01-02-01	Understand the principles of medical data management and storage, including electronic medical records, and comply with relevant regulations, laws, ethical standards, and provisions for protecting personal information.	**Q4**
IT-01-02-02	Understand and practice appropriate use of social media as a healthcare professional.	**Q5**
**IT-02: Principles of information science and technology necessary for medical care and surrounding society**		
IT-02-01: Medical care using information science and technology		
IT-02-01-01	Use digital devices, such as PCs and smartphones, to make use of information science technology, such as the internet and apps, in medical practice.	**Q6**
IT-02-01-02	Solve problems using the information and data collected using information science and technology.	**Q7**
IT-02-02: Knowledge of advanced information science and technology		
IT-02-02-01	Understand information science and technology related to medical care (medical information systems, wearable devices, applications, artificial intelligence, telemedicine technology, and the Internet of Things [IoT]) and discuss their potential applications.	**Q8**
IT-02-02-02	Understand the role required of medical professionals when applying information science and technology to medical care by working together with relevant specialists.	**Q9**
**IT-03: Application of information science and technology in clinical practice**		
IT-03-01: Communication skills using information science and technology		
IT-03-01-01	Demonstrate effective documentation and use of features unique to electronic medical records.	**Q10**
IT-03-01-02	Understand the pros and cons of remote communications, and select and use appropriate tools (e-mail, video conference systems, and social media) according to the intended purpose.	**Q11**
IT-03-02: Learning skills using information science and technology		
IT-03-02-01	Use appropriate digital devices and applications (e-learning, mobile technology, etc.) for self-learning and cooperative learning.	**Q12**
IT-03-02-02	Develop flexibility in using new information science and technology in one’s own learning and medical practice.	**Q13**

### Data collection

After obtaining permission from the 82 medical schools in Japan, we recruited participants for the survey. We mailed information about the study, along with a survey form to the schools. The faculty of each school distributed this information to sixth-year medical students. The students received a detailed research description that explained the purpose and significance of the study, outlined the survey items, and emphasized that participation was anonymous and voluntary.

### Data analysis

For the items on competencies necessary to use IST based on the fourth tier learning objectives, we examined the means and standard deviations, medians and interquartile ranges. Further, we used Cronbach’s alpha to assess internal consistency—an alpha coefficient of 0.7 or more indicating a sufficient level of consistency [25].

To examine the factors that improve the self-assessment of the fourth tier learning objectives, we conducted a logistic regression analysis in which self-assessment was divided into two values: “able/somewhat able” and “not able/not very able.” To examine the factors that improve the low self-assessment of competencies, the learning objectives of the fourth tier that had a higher percentage of “not able” and “not very able” were used as the dependent variables in the logistic regression analysis. We chose factors that were associated with self-assessment in the univariate analysis as the independent variables (with a criterion of *p* < 0.25) [26].

In this study, we considered *p* < 0.05 as statistically significant, and performed all statistical analyses using EZR. We used the graphical user interface of R (The R Foundation for Statistical Computing, Vienna, Austria; Saitama Medical Center, Jichi Medical University, Saitama, Japan) to perform the analyses [27]. A medical statistician (MM) validated all the statistical analyses.

### Ethical consideration

The study description stated that participation was voluntary and would not affect the students’ grades. Medical students who agreed to participate accessed the questionnaire.

The study was approved by the Ethics Review Committee of Jichi Medical University School of Medicine (reference number: 22–097).

## Results

### Demographic characteristics and pre- and post-medical school learning experiences

The 824 participants who agreed to participate in the study provided valid responses with no missing values for any questionnaire items. Of the participants, 63.7%, 35.2%, and 1.1% were male, female, and others, respectively. The median age was 25 years (IQR: 24–26). Of the participants, 50.9% were affiliated with a medical university. A total of 2.63% of participants had work experience in IST prior to medical school. Furthermore, 55.7% had learned IST in the classroom, and 23.7% had learned it outside the classroom before entering medical school. Participants who had started using IST in the classroom while they were in elementary school were the highest percentage (39.2%). In contrast, 44.5% of the respondents started using IST for self-study after entering medical school. In addition, 52.7% of the students attended problem-solving classes before entering medical school. Table 2 presents the percentage of students who attended classes on IST after entering medical school.

**Table 2:** Attendance rate for Information Science and Technology Classes among Medical School entrants

Experience taking classes *N*(%)	Yes	No
IT-01-01: Preparation for dealing with information, science, and technology	519 (62.99)	305 (37.01)
IT-01-02: Ethics and rules for using information science and technology	640 (77.67)	184 (22.33)
IT-02-01: Medical care using information science and technology	588 (71.36)	236 (28.64)
IT-02-02: Knowledge of advanced information science and technology	448 (54.37)	376 (45.63)
IT-03-01: Communication skills using information science and technology	481 (58.37)	343 (41.63)
IT-03-02: Learning skills using information science and technology	486 (58.98)	338 (41.02)

### Self-assessment of competencies required to use IST

To determine the reliability of the overall competencies required to use IST, we checked the correlation matrix among the items and calculated Cronbach’s alpha. None of the items had a correlation coefficient lower than 0.4 (Appendix 1). The Cronbach’s alpha coefficient for the overall competencies was 0.853, and no item (Appendix 2) had a Cronbach’s alpha coefficient lower than 0.9.

Table 3 presents the results of the self-assessment of competencies mentioned under the fourth tier learning objectives. The mean scores for IT-01-02-01 and IT-01-02-02 were the highest at 3.50 and 3.37 (SD 0.82 and 0.69). In contrast, more than 30% of the students answered “not able or not very able” on three items (IT-01-01-02, IT-01-01-03, and IT-02-02-01).

**Table 3:** Self-Assessment of Information Science and Technology Competencies for Fourth-Tier Learning objectives

Question items	*n* (%)				Mean (SD)	Median[IQR]
	able	somewhat able	not very able	not able		
IT01-01-01 Understand the importance and social significance of using information science and technology in medicine.	290 (35.19)	397 (48.18)	117 (14.20)	20 (2.43)	3.16 (0.75)	3 [3, 4]
IT01-01-02 Understand an overview of the regulations, laws, and guidelines related to information science and technology in medicine.	184 (22.33)	362 (43.93)	221 (26.82)	57 (6.92)	2.81 (0.86)	3 [2, 3]
IT01-01-03 Discuss ethical issues, such as social disparities caused by the digital divide, that may arise in the use of information science and technology in medicine.	154 (18.69)	396 (48.06)	220 (26.70)	54 (6.55)	2.79 (0.82)	3 [2, 3]
IT01-02-01 Understand the principles of medical data management and storage, including electronic medical records, and comply with relevant regulations, laws, ethical standards, and provisions for protecting personal information.	492 (59.71)	271 (32.89)	45 (5.46)	16 (1.94)	3.50 (0.69)	4 [3, 4]
IT01-02-02 Understand and practice appropriate use of social media as a healthcare professional.	420 (50.97)	301 (36.53)	89 (10.80)	14 (1.70)	3.37 (0.74)	4 [3, 4]
IT02-01-01 Use digital devices, such as PCs and smartphones, to make use of information science technology, such as the internet and apps, in medical practice.	271(32.89)	411 (49.88)	122 (14.81)	20 (2.43)	3.13 (0.75)	3 [3, 4]
IT02-01-02 Solve problems using the information and data collected using information science and technology.	267 (32.40)	423 (51.33)	111 (13.47)	23 (2.79)	3.13 (0.74)	3 [3, 4]
IT02-02-01 Understand information science and technology related to medical care (medical information systems, wearable devices, applications, artificial intelligence, telemedicine technology, and the Internet of Things [IoT]) and discuss their potential applications.	181 (21.97)	350 (42.48)	232 (28.16)	61 (7.40)	2.79 (0.87)	3 [2, 3]
IT02-02-02 Understand the role required of medical professionals when applying information science andtechnology to medical care by working together with relevant specialists.	174 (21.12)	432 (52.43)	190 (23.06)	28 (3.40)	2.91 (0.76)	3 [2, 3]
IT03-01-01 Demonstrate effective documentation and use of features unique to electronic medical records.	292 (35.44)	386 (46.84)	123 (14.93)	23 (2.79)	3.15 (0.77)	3 [3, 4]
IT03-01-02 Understand the pros and cons of remote communications, and select and use appropriate tools (e-mail, video conference systems, and social media) according to the intended purpose.	274 (33.25)	415 (50.36)	118 (14.32)	17 (2.06)	3.15 (0.73)	3 [3, 4]
IT03-02-01 Use appropriate digital devices and applications (e-learning, mobile technology, etc.) for self-learning and cooperative learning.	309 (37.50)	372 (45.15)	112 (13.59)	31 (3.76)	3.16 (0.80)	3 [3, 4]
IT03-02-02 Develop flexibility in using new information science and technology in one’s own learning and medical practice.	238 (28.88)	419 (50.85)	147 (17.84)	20 (2.43)	3.06 (0.75)	3 [3, 4]

### Relevant factors affecting the learning objectives related to competencies self-assessed as low

We conducted a logistic regression analysis using the self-assessments of IT-01-01-02, IT-01-01-03, and IT-02-02-01 as dependent variables (Table 4). We did not consider gender with a variance inflation factor value greater than 2 as an independent variable because of multicollinearity (Appendix 3).

**Table 4:** Logistic Regression Analysis for Information Science and Technology Competencies Self-Assessments

	IT-01-01-02: Understand an overview of the regulations, laws, and guidelines related to information science and technology in medicine					IT-01-01-03: Discuss ethical issues, such as social disparities caused by the digital divide, that may arise in the use of information science and technology in medicine					IT-02-02-01: Understand information science and technology related to medical care (medical information systems, wearable devices, applications, artificial intelligence, telemedicine technology, and the Internet of Things [IoT]) and discuss their potential applications				
	β	SD	*p*-value	OR	95%CI	β	SD	*p*-value	OR	95%CI	β	SD	*p*-value	OR	95%CI
Intercept	-0.8762	0.5868	0.14	0.42	(0.13-1.32)	0.1511	0.6915	0.83	1.16	(0.30-4.51)	-0.0804	0.6960	0.91	0.92	(0.24-3.61)
Experience taking classes IT-01-01: Preparation for dealing with information, science, and technology	0.4838	0.2041	0.02	1.62	(1.09-2.42)	0.1689	0.2049	0.41	1.18	(0.79-1.77)	0.1354	0.2069	0.51	1.15	(0.76-1.72)
Experience taking classes IT-01-02: Ethics and rules for using information science and technology	0.2756	0.2315	0.23	1.32	(0.84-2.07)	0.3440	0.2310	0.14	1.41	(0.90-2.22)	-0.1979	0.2391	0.41	0.82	(0.51-1.31)
Experience taking classes IT-02-01: Medical care using information science and technology	-0.1250	0.2290	0.58	0.88	(0.56-1.38)	-0.2057	0.2281	0.37	0.81	(0.52-1.27)	0.6075	0.2262	> 0.01	1.84	(1.18-2.86)
Experience taking classesIT-02-02: Knowledge of advanced information science and technology	0.2107	0.2254	0.35	1.23	(0.79-1.92)	0.4467	0.2209	0.04	1.56	(1.01-2.41)	0.8501	0.2179	> 0.001	2.34	(1.53-3.59)
Experience taking classes IT-03-01: Communication skills using information science and technology	0.7918	0.2286	> 0.001	2.21	(1.41-3.46)	0.3653	0.2285	0.11	1.44	(0.92-2.25)	0.6853	0.2286	> 0.01	1.98	(1.27-3.11)
Experience taking classes IT-03-02: Learning skills using information science and technology	0.5145	0.2344	0.03	1.67	(1.06-2.65)	0.6766	0.2331	> 0.01	1.97	(1.25-3.11)	-0.1712	0.2404	0.48	0.84	(0.53-1.35)
Experience with problem-solving classes	0.3614	0.1696	0.03	1.44	(1.03-2.00)	0.3460	0.1677	0.04	1.41	(1.02-1.96)	0.3205	0.1684	0.057	1.38	(0.99-1.92)
First used information science and technology outside of school	0.1854	0.2081	0.37	1.2	(0.80-1.81)	0.3971	0.2099	0.06	1.49	(0.99-2.24)	0.7440	0.2166	> 0.001	2.1	(1.38-3.22)
First used information science and technology inside of school	0.1501	0.1713	0.38	1.16	(0.83-1.63)	0.0888	0.1691	0.60	1.09	(0.78-1.52)	-0.0078	0.1702	0.96	0.99	(0.71-1.39)
Any work experience with information, science, and technology	0.0795	0.5513	0.88	1.08	(0.37-3.19)	-0.8197	0.6655	0.22	0.44	(0.12-1.62)	-0.7271	0.6689	0.28	0.48	(0.13-1.79)
Nagelkerke R²	0.2194					0.1923					0.2195				

*The independent variable is set to 1 for all items and 0 for none

*The dependent variable was classified into two values, “able/somewhat able” and “not very able/not able”

In the model that examined IT-01-01-02, the experience of taking classes IT-03-01 and IT-03-02, as well as problem-solving classes, was statistically significant. In the model that examined IT-01-01-03, the factors experience of taking classes IT-02-02 and IT-03-02, and experience with problem-solving classes were statistically significant. Similarly, in the model examining IT-01-01-03, the factors experience of taking classes IT-02-01, IT-02-02, IT-03-01, and first use of information science and technology outside of school were statistically significant.

To evaluate the goodness-of-fit of the logistic regression models, we used Nagelkerke’s pseudo R-squared (Nagelkerke R²). The logistic regression analysis showed that the Nagelkerke R² values were 0.2194 for IT-01-01-02, 0.1923 for IT-01-01-03, and 0.2195 for IT-02-02-01, indicating that the analyses for IT-01-01-02 and IT-02-02-01 had similar explanatory power, each explaining approximately 21.94% and 21.95% of the variance respectively. The analysis for IT-01-01-03 had a slightly lower explanatory power, explaining about 19.23% of the variance.

## Discussion

We conducted this cross-sectional investigation to examine the extent to which Japanese medical students perceive that they have acquired competencies to use IST upon completion of the curriculum, and the factors that may promote or hinder such acquisition.

We found that (1) medical students’ self-assessed certain competencies as high and others as low. Those with particularly low self-assessment included “Understand an overview of the regulations, laws, and guidelines related to information science and technology in medicine” (IT-01-01-02), “Discuss ethical issues, such as social disparities caused by the digital divide, that may arise in the use of information science and technology in medicine” (IT-01-01-03), and “Understand information science and technology related to medical care (medical information systems, wearable devices, applications, artificial intelligence, telemedicine technology, and the Internet of Things [IoT]) and discuss their potential applications” (IT-02-02-01). (2) The factor associated with improvement in the three self-assessed lower learning objectives (IT-01-01-02, IT-01-01-03, and IT-02-02-01) was the experience of taking a specific IST class at the medical school. Additionally, we found that for IT-01-01-02 and IT-01-01-03, participation in problem-solving classes before medical school was associated with improved self-assessment. For IT-02-02-01, the experience of learning IST outside formal classes before entering medical school correlated with higher self-assessment.

### Factors correlated to IST-related subcompetencies pertaining to regulatory and ethical issues

IT-01-01-02 and IT-01-01-03, which define subcompetencies addressing regulatory and ethical issues related to IST in medicine, were associated with experiences with problem-based learning prior to medical school and experience with IT-03-02 classes (learning skills using IST in medicine classes in medical school). As IST is constantly evolving, the relevant regulations are changing [28], and in Japan, the Act on the Protection of Personal Information in Relation to Data Privacy [29] is revised in principle every three years. Medical students must adopt a learning strategy that responds to rapid changes, and they must learn to apply the knowledge and skills they have acquired. Therefore, they must be metacognizant of their own knowledge and skills in IST and explore learning issues on their own. This learning process is consistent with the problem-solving learning framework [30]. The reason for the low self-evaluation of these two academic objectives may be related to the fact that the participants had little experience with problem-solving-based learning before entering medical school. Although Japan’s courses of study have been repeatedly revised since 1990 based on the standard of being able to spontaneously identify problems, think independently, make judgments, and express oneself, the 2010s did not see a departure from rote learning [31]. Therefore, participants in this study may have lacked experience with problem-based learning before entering medical school; in fact, 47% of the students had no experience with problem-based learning. Furthermore, a survey conducted by the Ministry of Education, Culture, Sports, Science and Technology indicated that even for 53% of students with experience, many were not good at problem-solving learning when they enrolled in junior high school [32]. In other words, they were unable to set their own tasks and learn about the rapidly changing regulations and ethical issues related to IST; consequently, their self-assessment was low.

### Factors correlated to subcompetencies pertaining to IST use in medical practice

The results suggest that the low self-assessment of IT-02-02-01 is related to a lack of learning opportunities associated with IST in medicine within the curriculum. We found that students had the least experience with the IT-02-02 (advanced knowledge of IST class), but their experience with this class was associated with an improvement in the self-evaluation of IT-02-02-01. The correlation between the self-assessment of IT-02-02-01 and the “experience of learning IST outside of regular classes before entering medical school” suggests that the habit of self-learning content that cannot be learned through the MCC is essential for acquiring knowledge regarding IST in medical care. Among the participants, 24% had learned IST outside the formal curriculum before entering medical school, which may have resulted in the low self-assessment of IT-02-02-01.

Thus, learning experience within the curriculum is one of the most important factors in acquiring knowledge on IST in medicine, and therefore, future studies should re-examine whether IT-02-02-01 can be improved by a curriculum based on the MCC 2022 Revision. However, reports from other countries state that computer literacy indicators for medical students focus on software use and Internet safety [21, 22]. These studies assume that there is a lack of learning experience related to IST in medicine within the curriculum. Additionally, as IST is constantly evolving, maintaining an up-to-date curriculum that comprehensively covers the subject can be challenging. Establishing fundamental competencies for active learning for medical students and enabling them to continuously learn about IST on their own may be an essential improvement measure for acquiring these competencies.

### Limitation and future research plan

To the authors’ knowledge, this study is the first to describe medical students’ self-assessment of competencies related to the use of IST and the factors that affect this assessment. The results of this study are expected to be used for future curriculum management of competencies related to IST.

However, this study has several limitations. First, it was conducted only among Japanese medical students. This may affect the results’ generalizability because medical schools in Japan follow a six-year pattern and medical students extensively use IST in their daily lives. Second, this study was conducted over a single year; thus, it is possible that events that occurred in FY2022 may have affected the results. Finally, the survey on the acquisition of competencies to use IST was based on self-assessment by medical students. It is possible that the students’ responses contained cognitive biases, which questions the credibility of the self-assessment.

In the future, we expect that countries other than Japan will conduct surveys on medical students’ acquisition of competencies required to use IST, and the factors that affect their self-assessment. By comparing the results from other countries, we can determine the educational systems and cultural backgrounds that positively influence the acquisition of such competencies. Next, it is desirable to survey the actual status of acquisition of competencies required to use IST over several years. In this study, we found a correlation between the self-assessment of IST and the participants’ learning experience. Therefore, it is important to avoid bias in the responses related to factors that affect participants’ self-assessment. Finally, the competencies required to use IST should be examined objectively. Because self-assessed competencies are based on subjective responses from medical students, a third party should observe and assess medical students’ competencies required to use IST. No effective indicator can objectively measure the competencies required to use IST [6]; thus, we believe that further development is required to clarify the results.

## Conclusion

We conducted a survey to identify the factors that improve Japanese medical students’ self-assessment of their acquisition of competencies required to use IST. The results showed that, among the learning objectives, medical students self-assessed their lack of mastery of regulatory and ethical issues related to IST, as well as their understanding of IST in medical care as low. The factors that enhanced medical students’ self-assessment were their experience of taking classes on IST, their experience of problem-based learning and learning IST outside formal classes, and the way they learn about IST. However, the rapidly evolving IST and the corresponding changes in regulation and curriculum have mandated that medical students adapt to the dynamic nature of IST. Therefore, we believe that the first step toward using IST in medicine is the recognition of the importance for medical students to learn about IST, adopt active learning methods as learners, and have experience in applying the skills they have acquired. We expect that the findings of this study will enhance medical education researchers’ awareness of the actual situation regarding students’ acquisition of competencies necessary to use IST and will facilitate the development of more effective interventions.

### Electronic supplementary material

Below is the link to the electronic supplementary material.

Supplementary Material 1

Supplementary Material 2

Supplementary Material 3

## Data Availability

The datasets generated and/or analysed during the current study are not publicly available due to ethical constraints but are available from the corresponding author on reasonable request.

## Abbreviations

COVID-19: Coronavirus disease
IST: Information science and technology
MCC: Model core curriculum

## References

[1] World Health Organization. Recommendations on digital interventions for health system strengthening: executive summary. World Health Organization. 2019. https://apps.who.int/iris/handle/10665/311977. Accessed October 21 2023.

[2] Majeed A, Maile EJ, Bindman AB. The primary care response to COVID-19 in England’s National Health Service. J R Soc Med. 2020;113:208–10. 32521196 10.1177/0141076820931452 PMC7439588

[3] Nazeha N, Pavagadhi D, Kyaw BM, Car J, Jimenez G, Tudor Car L. A digitally competent health workforce: scoping review of educational frameworks. J Med Internet Res. 2020;22:e22706. 33151152 10.2196/22706 PMC7677019

[4] Wong BLH, Khurana MP, Smith RD, El-Omrani O, Pold A, Lotfi A, et al. Harnessing the digital potential of the next generation of health professionals. Hum Resour Health. 2021;19:50. 10.1186/s12960-021-00591-2. https://human-resources-health.biomedcentral.com/articles/. 33853625 10.1186/s12960-021-00591-2 PMC8045994

[5] Chen Y, Banerjee A. Improving the digital health of the workforce in the COVID-19 context: an opportunity to future-proof medical training. Future Healthc J. 2020;7:189–92. 10.7861/fhj.2020-0162. https://www.rcpjournals.org/lookup/doi/. 33094221 10.7861/fhj.2020-0162 PMC7571768

[6] Longhini J, Rossettini G, Palese A. Digital health competencies among health care professionals: systematic review. J Med Internet Res. 2022;24:e36414. 35980735 10.2196/36414 PMC9437781

[7] Konttila J, Siira H, Kyngäs H, Lahtinen M, Elo S, Kääriäinen M, et al. Healthcare professionals’ competence in digitalisation: a systematic review. J Clin Nurs. 2019;28:745–61. 30376199 10.1111/jocn.14710

[8] HITComp. Health information technology competencies (HITComp). EU*US ehealth work project. 2015. http://hitcomp.org/competencies/. Accessed November 27 2023.

[9] Canada Health Infoway and association of faculties of medicine of Canada. Ehealth competencies for undergraduate medical education. 2014. http://www.ehealthresources.ca/sites/default/files/pdf/eHealth%20Competencies%20for%20UME.pdf. Accessed November 27 2023.

[10] Machleid F, Kaczmarczyk R, Johann D, Balčiūnas J, Atienza-Carbonell B, von Maltzahn F, et al. Perceptions of digital health education among European medical students: mixed methods survey. J Med Internet Res. 2020;22:e19827. https://www.jmir.org/2020/8/e19827. 32667899 10.2196/19827 PMC7455864

[11] Gagnon MP, Légaré F, Labrecque M, Frémont P, Pluye P, Gagnon J, et al. Interventions for promoting information and communication technologies adoption in healthcare professionals. Cochrane Database Syst Rev. 2009;1:CD006093. 10.1002/14651858.CD006093.pub2 PMC3973635 19160265

[12] Khurana MP, Raaschou-Pedersen DE, Kurtzhals J, Bardram JE, Ostrowski SR, Bundgaard JS. Digital health competencies in medical school education: a scoping review and Delphi method study. BMC Med Educ. 2022;22:129. 35216611 10.1186/s12909-022-03163-7 PMC8881190

[13] Biggins D, Holley D, Zezulkova M. Digital Competence and Capability Frameworks in Higher Education: Importance of Life-long Learning, Self-Development and Well-being. EAI Endorsed Transactions on e-Learning;4.

[14] Association of American Medical Colleges. Core EPAs guiding principles. Core entrustable professional activities for entering residency curriculum developers’ guide. https://store.aamc.org/downloadable/download/sample/sample_id/63/%20. Accessed October 21 2023.

[15] General Medical Council. Outcomes for graduates 2018; 2018. https://www.gmc-uk.org/-/media/documents/dc11326-outcomes-for-graduates-2018_pdf-75040796.pdf. Accessed October 21 2023.

[16] The Royal College of Physicians and Surgeons of Canada. CanMEDS 2015 physician competency framework. 2015. https://canmeds.royalcollege.ca/uploads/en/framework/CanMEDS%202015%20Framework_EN_Reduced.pdf. Accessed October 21 2023.

[17] National Medical Undergraduate Curriculum Committee. Outcomes and standards for undergraduate medical education in Singapore. 2014. https://www.moh.gov.sg/docs/librariesprovider4/guidelines/nmucc_report_singlepage1bc789a9a9004d11acc61e169e671da5.pdf. Accessed October 21 2023.

[18] Nederladse federatie van universitair medische centra. 2020. Medical Training Framework. https://www.nfu.nl/sites/default/files/2020-08/20.1577_Raamplan_Medical_Training_Framework_2020_-_May_2020.pdf. Accessed October 21 2023.

[19] Medical education model core curriculum expert Research Committee. The model core curriculum for medical education in Japan 2022 revision. https://www.mext.go.jp/content/20230315-mxt_igaku-000026049_00003.pdf. Accessed October 21 2023.

[20] Medical education model core curriculum expert Research Committee. The model core curriculum for medical education in Japan 2017 revision. https://www.mext.go.jp/component/b_menu/shingi/toushin/__icsFiles/afieldfile/2017/06/28/1383961_01.pdf. Accessed October 21 2023.

[21] Morton CE, Smith SF, Lwin T, George M, Williams M. Computer programming: should medical students be learning it? JMIR Med Educ. 2019;5:e11940. 30901000 10.2196/11940 PMC6450476

[22] Tudor Car L, Kyaw BM, Nannan Panday RS, van der Kleij R, Chavannes N, Majeed A, et al. Digital health training programs for medical students: scoping review. JMIR Med Educ. 2021;7:e28275. 34287206 10.2196/28275 PMC8339984

[23] Faul F, Erdfelder E, Lang AG, Buchner A. G*Power 3: a flexible statistical power analysis program for the social, behavioral, and biomedical sciences. Behav Res Methods. 2007;39:175–91. 17695343 10.3758/BF03193146

[24] Cohen J. Statistical power analysis for the behavioral sciences. 2nd ed. Hillsdale, NJ: Lawrence Erlbaum; 1988.

[25] Hulley SB, Cummings SR, Browner WS, Grady DG, Newman TB. Designing clinical research. 4th ed. Philadelphia: Lippincott Williams & Wilkins; 2015. p. 341.

[26] Hosmer WD, Lemeshow S. Applied logistic regression. 2nd ed. New York: A Wiley Interscience Publication; 2000.

[27] Kanda Y. Investigation of the freely available easy-to-use software ‘EZR’ for medical statistics. Bone Marrow Transpl. 2013;48:452–8. 10.1038/bmt.2012.244 PMC3590441 23208313

[28] Vincent CJ, Niezen G, O’Kane AA, Stawarz K. Can standards and regulations keep up with health technology? JMIR MHealth UHealth. 2015;3:e64. 10.2196/mhealth.3918 PMC4526895 26041730

[29] Ministry of internal affairs and communications. 2003. Act on the Protection of Personal Information. https://www.japaneselawtranslation.go.jp/ja/laws/view/130. Accessed November 27 2023.

[30] Taylor D, Miflin B. Problem-based learning: where are we now? Med Teach. 2008;30:742–63. 18946818 10.1080/01421590802217199

[31] Yamanaka S, Suzuki KH. In: Reimers FM, editor. Japanese education reform towards twenty-first century education. Audacious Education Purposes. Springer; 2020.

[32] Ministry of Education. Culture, Sports, Science and Technology. Information literacy survey cooperation committee [Japanese]. https://www.mext.go.jp/a_menu/shotou/zyouhou/detail/__icsFiles/afieldfile/2017/01/18/1381046_02_1.pdf. Accessed November 27 2023.

